# Molecular identification of *Haemonchus contortus* in sheep from Upper Egypt

**DOI:** 10.3389/fvets.2023.1327424

**Published:** 2024-02-12

**Authors:** Sara Abdel-Aal Mohamed, Ahmed Kamal Dyab, Enrique Raya-Álvarez, Fatma Mohamed Abdel-Aziz, Fathy Osman, Ahmed Gareh, Alshimaa M. M. Farag, Doaa Salman, Manal F. El-Khadragy, Daniel Bravo-Barriga, Ahmad Agil, Ehab Kotb Elmahallawy

**Affiliations:** ^1^Department of Parasitology, Faculty of Veterinary Medicine, Assiut University, Assiut, Egypt; ^2^Department of Parasitology, Faculty of Medicine, Assiut University, Assiut, Egypt; ^3^Rheumatology Department, Hospital Universitario San Cecilio, Av. de la Investigación, Granada, Spain; ^4^Department of Parasitology, Animal Health Research Institute, Agriculture Research Center (ARC), Giza, Egypt; ^5^Department of Parasitology, Faculty of Veterinary Medicine, Aswan University, Aswan, Egypt; ^6^Department of Internal Medicine and Infectious Diseases Faculty of Veterinary Medicine, Mansoura University, Mansoura, Egypt; ^7^Department of Animal Medicine, Faculty of Veterinary Medicine, Sohag University, Sohag, Egypt; ^8^Department of Biology, College of Science, Princess Nourah bint Abdulrahman University, Riyadh, Saudi Arabia; ^9^Departamento de Sanidad Animal, Grupo de Investigación en Salud Animal y Zoonosis (GISAZ), UIC Zoonosis y Enfermedades Emergentes (ENZOEM), Facultad de Veterinaria, Universidad de Córdoba, Córdoba, Spain; ^10^Department of Pharmacology, Biohealth Institute Granada (IBs Granada) and Neuroscience Institute, School of Medicine, University of Granada, Granada, Spain; ^11^Department of Zoonoses, Faculty of Veterinary Medicine, Sohag University, Sohag, Egypt

**Keywords:** haemonchosis, morphological, molecular, sheep, Upper Egypt

## Abstract

**Introduction:**

*Haemonchus* spp. are considered the most important strongylid nematodes with a worldwide distribution. The parasite’s blood-sucking nature can lead to severe anemia in infected animals. Despite its widespread impact, there is a dearth of comprehensive data on morphological and molecular identification methods for *Haemonchus* spp. in sheep from Upper Egypt. To address this gap, our current study aimed to assess the prevalence of *Haemonchus* spp. in 400 sheep fecal samples.

**Methods:**

We employed microscopic examination and molecular techniques, using polymerase chain reaction (PCR) targeting the 18S gene for precise identification. Additionally, the potential risk factors associated with the infection by the parasite in sheep were explored.

**Results:**

The study pointed out that 33.00% (132 of 400) of the examined sheep were infected with *Haemonchus* spp. Sheep age and seasonal variability were found to be significant factors (*p* < 0.05) associated with the infection. Notably, sheep under 2 years old exhibited a higher risk, with an infection rate of 43.75% (84 out of 192), than their older counterparts. Furthermore, all reported infections were exclusively observed during the cold season, constituting 58.93% (132 out of 224) of cases. By contrast, no statistically significant association (*p* > 0.05) was found between the sex of the examined sheep and the occurrence of haemonchosis. Employing molecular methods, we isolated and identified the parasite through PCR analysis of cultured larvae, which were then subsequently confirmed as Haemonchus contortus via phylogenetic analysis.

**Discussion:**

The study concluded that there was a relatively high occurrence of *H. contortu*s among sheep from Upper Egypt. We recommend the implementation of stringent and effective control measures to combat the infection and safeguard livestock health.

## 1 Introduction

Sheep are considered one of the most important small ruminant livestock globally (1). They represent a major source of wool, meat, milk, and skins, which together form a vital component of the rural economy, mainly in arid and semi-arid areas (2). In Egypt, sheep and goats are among the most important animals for meeting the requirements for meat for human consumption (3). However, this industry is subjected to a wide variety of parasitic infections that affect the health status of animals, causing great losses in the livestock industry (4). Among others, infection of sheep by gastrointestinal parasites (GIP) is one of the major health problems in the sheep industry, leading to a loss of plasma protein, alterations in protein metabolism, diarrhea, and a loss of body weight. Additionally, the infection by these parasites suppresses the immune system of the infected animals, which in turn makes them more susceptible to other pathogenic agents (5).

Haemonchosis is regarded as a highly destructive disease that significantly reduces the productivity of infected sheep and has substantial economic consequences. This includes elevated levels of morbidity and mortality and increased management costs associated with implementing control measures (6, 7). The disease is caused by *Haemonchus* spp. or barber’s pole worm, which is considered a principal abomasal worm of ruminants with a worldwide distribution. Nearly 12 species have been recognized in domestic ruminants (8). Among others, *Haemonchus contortus* is considered the nematode of greatest economic importance in small ruminants (9) and extremely pathogenic in sheep (10). Subclinical infection with *H. contortus* can lead to reduced weight gain and appetite. Moreover, heavy infection by these worms might result in the progression of various clinical signs that involve weight loss, submandibular edema, and diarrhea (11). Additionally, these parasites are voracious blood suckers, and therefore, heavy infection by the parasite can cause lethal anemia and might lead to death in heavily infected animals (12). Given its veterinary importance with regard to the health and productivity of sheep, periodical updates about the disease at a national level is very important for providing effective control measures for the disease.

Taken into account, *Haemonchus* spp. has a direct life cycle. The adult female inhabits the abomasum or intestine and then immature eggs pass in the stool. Later, immature eggs develop in the external environment and hatch to give first-stage larva (L1). Larvae nourishment is mainly based on bacteria, and then they molt to the second larval stage (L2) and undergo another molt to reach the infective third larval stage (L3). Sheep contract the infection through third-stage larvae (infective stage) during grazing, which then reach the final predilection site and develop to L5 (13). Environmental conditions, mainly temperature and humidity, influence the survival of the larvae of *Haemonchus* (14, 15). In this regard, cool and humid climatic conditions favor the survival of L3 for several months. By contrast, L3 might survive for a shorter time in warm weather due to the higher metabolic rate that speedily reduces energy reserves (16, 17). The identification of *Haemonchus* spp. could be achieved by several approaches, including direct fecal smears, fecal flotation for detecting parasitic eggs, the rearing of eggs in culture to obtain L3, and postmortem examination to determine immature and adult worms (18). However, these methods have low sensitivity and lack the ability to distinguish the circulating species/genotypes. On the other hand, fecal culture methods for the identification of nematode larvae followed by the screening of positive samples using PCR-based techniques possess many advantages and might help confirm the infection (19). As shown in Table 1, few epidemiological and molecular studies have explored the occurrence of *Haemonchus* spp. circulating in Sheep in Egypt, particularly in the upper part of the country (19–36). Based on information provided by previous studies, this study was undertaken to morphologically and molecularly identify *Haemonchus* in sheep. Additionally, the research included a comprehensive review of previous studies on the various *Haemonchus* species circulating among sheep in Egypt.

**Table 1:** Occurrence and genetic diversity of *Haemonchus* spp. reported in sheep in Egypt.

Area	Detection method	Frequency % (no. pos./total)	Species identified	Genotype (no.)	Reference
Upper Egypt	PM	11.50 (36/312)	*H. contortus*	ND	(20)
Lower Egypt	PM	07.90 (15/189)	*H. contortus*	ND	(21)
Lower Egypt	CM	30.17(35/116)	*Haemonchus* spp.	ND	(22)
Upper Egypt	CM&PM	18.00 (27/150) 49.06 (26/53)	*H. contortus*	ND	(23)
Lower Egypt	CM&PM	30.00 (95/319)	*Haemonchus* spp.	ND	(24)
Upper Egypt	CM	16.10 (196/1217)	*H. contortus*	ND	(25)
Lower Egypt	CM	14.20 (118/830)	*Haemonchus* spp.	ND	(26)
Upper Egypt	CM	23.62 (232/982)	*Haemonchus* sp.	ND	(27)
Upper Egypt	ELISA	00.00 (0/880)	*H. contortus*	ND	(28)
Lower Egypt	CM	43.30 (39/90)	*H. contortus*	ND	(29)
Lower Egypt	ELISA	44.62 (123/275)	*Haemonchus* spp.	ND	(30)
Upper Egypt	CM & FC	33.75 (303/900)	*Haemonchus* spp.	ND	(31)
Upper Egypt	CM & FC	26.00 (19/72)	*Haemonchus* spp.	ND	(32)
Lower Egypt	CM & PM	*H. contortus* 3.5 (6/173) *H. placei* 1.7% (3/173)	*H. contortus* *H. placei*	ND	(33)
Lower Egypt	PCR	26.50% (173/653)	*H. contortus*	ND	(34)
Upper Egypt	CM&ELISA	27.10 (29/107) 64.48 (69/107)	*H. contortus*	ND	(35)
Upper, Lower Egypt	PCR	ND	*H. contortus*	ND	(19)
Upper Egypt	CM & FC	17.83 (28/157)	*H. contortus*	ND	(36)

CM, Conventional Microscope; FC, Fecal Culture; PM, Postmortem; PCR, Polymerase Chain Reaction; ELISA, Enzyme Linked Immunosorbent Assay; ND, Not detected.

## 2 Materials and methods

### 2.1 Ethical considerations

The study procedures obtained the approval of the research ethical committee of the Faculty of Veterinary Medicine, Assiut University (approval number, 06/2023/0096).

### 2.2 Study area and sample collection

**Figure 1:**
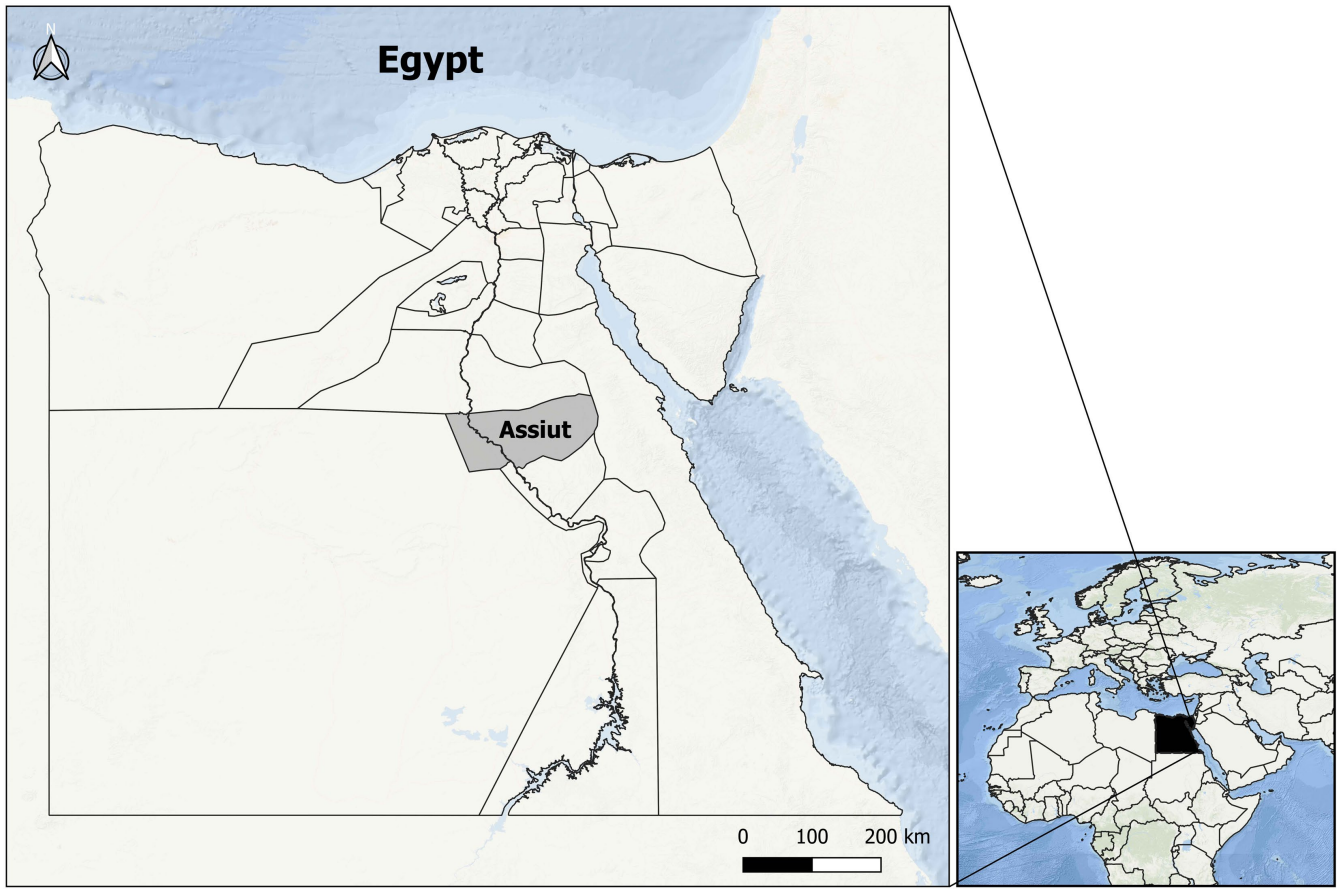
Map of Egypt where the study area, Asyut Governorate, is located (in gray).

The present study was conducted from February 2022 to January 2023 in Assiut Governorate, as depicted in Figure 1. A total of 400 rectal fecal samples were collected from sheep owned by small stakeholders participating in veterinary campaigns in Assiut Governorate. Each sample, ranging from 1 to 6 g in size, was collected in a clean, dry, sterile, and labeled screw top plastic cup. Relevant information, such as the animals’ sex, age, and date of sample collection, was recorded. Subsequently, the samples were transported to the Parasitology Laboratory at the Faculty of Veterinary Medicine (Assiut University, Egypt) for further processing and examination.

### 2.3 Macroscopic and microscopic examination of feces

The collected samples were carefully examined by the naked eye for their consistency and color, the presence of blood, mucus, and other unusual elements, and the existence of adult parasites and/or segments.

#### 2.3.1 Simple salt floatation technique

This technique was performed as described by Kaufmann (37). The fecal samples from the sheep were placed in a cup and then mixed thoroughly with saline solution. The mixture was poured through a wire mesh screen to remove large lumps. The suspension was transferred to a conical measure, filled with saline solution to the top, and allowed to settle for 30 min. The supernatant was discarded carefully and the remaining sediment (10 mL) was stirred, and a 2-ml sample was poured into a centrifuge tube. Then, saturated NaCl was added until a convex meniscus appeared at the top of the tube. Later, a square cover glass (19 × 19 mm) was placed on the tube. The tube was centrifuged at 2,000 rpm for 2–3 min. The cover glass was removed and the sample was placed on a slide and examined microscopically.

#### 2.3.2 Ordinary sedimentation technique

This step was performed according to the method described by Urquhart et al. (38). Briefly, fecal material was mixed with saline solution (0.90%), strained through a 40 μm nylon sieve, and then allowed to settle for 30 min, after which the supernatant was decanted. The sediment was resuspended in saline solution, after which washing was repeated until a clear supernatant was obtained. A drop of the sediment was pipetted onto a glass slide, covered with a cover slip, and then examined using a microscope (10×).

#### 2.3.3 The formalin ether concentration technique

This step was carried out as described by Balakrishna et al. (39). Fecal samples were emulsified in water (10 mL), strained through two layers of gauze, and allowed to settle for 30 min, after which the supernatant fluid was decanted. The sediment was then resuspended in saline solution, followed by washing until a clear supernatant was obtained. Later, 10.00% formal saline (7 mL) was added to the sediment and allowed to stand for 30 min. Then, ether (3 mL) was added, and the tube was shaken vigorously and centrifuged at 2,000 rpm for 2 min, after which three layers formed. The three layers of the supernatant were poured off, and the sediment was used for slide preparation and examined microscopically.

#### 2.3.4 Fecal culture methods for the identification of nematode larvae

This step was performed according to the protocol described by Zajac and Garza (40). Briefly, the moist fecal samples were disintegrated in a container using a spatula, until the required consistency was obtained. The sample container was closed and placed in an incubator at 27 °C for 7–10 days to obtain the larval stage. Water was added to the cultures approximately every 1–2 days if the mixture became too dry, and the cultures were exposed to air daily. Then, the larvae were recovered using the Baermann technique and examined and identified with a light microscope (13, 41).

### 2.4 PCR and phylogenetic analysis

This step involved the extraction of DNA from larvae samples following the manufacturer’s protocol supplied with a QIAamp DNA mini kit. Negative controls were included during DNA extraction to rule out any potential contamination. The primers were designed to amplify a specific internal fragment of the 18S gene, which is approximately 900 base pairs long. In accordance with the protocol (42), the forward primer attached to a location approximately 100 base pairs inward from the 5′ end of the gene, while the reverse primer bound at a site approximately 700 base pairs inward from the 3′ end. The detailed information on primers sets and the cycling conditions during cPCR are shown in Supplementary Tables S1, S2. The reaction mixture (25 μL) consisted of 12.5 μL of Emerald Amp GT PCR mastermix (2x premix; Code No. RR310A), 1 μm of each primer, and 5 μL of DNA template. Millipore water was added to a total volume of 25 μL. PCR products were size fractionated by electrophoresis in 2.00% agarose gels stained with 6 μL GreenSafe (10 mg/mL) for each 100 mL gel. PCR products were purified using a QIAquick PCR Product extraction kit (Qiagen, Valencia). A Bigdye Terminator V3.1 cycle sequencing kit (PerkinElmer) was used for the sequence reaction, which was then purified using a Centrisep spin column. PCR products linked to positive amplifications were purified and sequenced using an Applied Biosystems 3,130 genetic analyzer (HITACHI, Japan). Species-level identity was obtained considering a > 99% identity score using BLASTn (Mega-BLASTn option).^1^ Only one nucleotide sequence obtained during this study was submitted to the NCBI database under the accession number OP984151 (43). Multiple alignments were carried out using MAFFT version 7 (44). For maximum likelihood (ML) phylogenetic analyses, the choice of the best-fitting evolutionary model was based on those defined using JModeltest2 (45) on the basis of the Akaike information criterion. Tree construction was carried out using Mega 11 (46). The evolutionary history was inferred by using the ML method and Tamura 3-parameter model (47). The tree with the highest log likelihood (−6790.54) is shown. The percentage of trees in which the associated taxa clustered together is shown next to the branches. Initial tree(s) for the heuristic search were obtained automatically by applying the Neighbor-Joining and BioNJ algorithms to a matrix of pairwise distances estimated using the Tamura 3 parameter model and then selecting the topology with a superior log likelihood value. A discrete Gamma distribution was used to model evolutionary rate differences among sites [5 categories (+G, parameter = 0.8209)]. The tree was drawn to scale, with branch lengths measured in the number of substitutions per site. This analysis involved 36 nucleotide sequences. There was a total of 1,405 positions in the final dataset. Bootstrap coefficients were calculated for 1,000 replicates, and only those with >70% support are shown in the tree. The phylogenetic trees were manipulated for display using FigTree v.1.4.2 (48–50).

### 2.5 Statistical analysis

The resulting data were analyzed using Statistical Package for Social Sciences (SPSS; version 26). The qualitative variables were recorded and compared using a chi-square test. The prevalence of *Haemonchus* in sheep were estimated from the ratio of positives to the total number of samples, with the exact binomial confidence intervals of 95% based on the score method, which is derived from (51). Logistic regression analyses were used to determine crude and adjusted odds ratios (OR) with corresponding 95% confidence intervals (95% CI) for factors associated with haemonchosis infection. A *p*-value <0.05 was significant and *p*-values <0.01 were highly significant.

## 3 Results

### 3.1 Prevalence of *Haemonchus* spp. in the fecal samples of examined sheep and risk factors

In this study, the overall prevalence of *Haemonchus* spp. in examined fecal samples from sheep was 33.00% (CI: 28.57–37.75; 132 of 400). Regarding age as a potential risk factor associated with the infection (Table 2), *Haemonchus* was most prevalent in sheep aged <2 years (43.75%; CI: 36.92–50.82) compared with those aged >2 years (23.08%; CI: 17.87–29.26; *p* = 0.028). Furthermore, the infection rate was higher in male animals (36.67%; CI: 28.58–45.58) than in females (31.43%; CI: 26.27–37.09) but no significant deviation (*p* > 0.05) was found. Additionally, the prevalence of *Haemonchus* spp. was significantly higher (*p* < 0.001) during the cold season, reaching 58.93% (CI: 52.39–65.17; Table 2).

**Table 2:** Prevalence of *Haemonchus* in fecal samples of the examined sheep in relation to sex, age, and season.

Variable	Total examined	Infected (%)	95% CI	OR (95% CI)	*p* value
Age					
< 2 years	192	84 (43.75)	36.92–50.82	2.59 (1.68–3.99)	0.028^*^
> 2 years	208	48 (23.08)	17.87–29.26		
Sex					
Male	120	44 (36.67)	28.58–45.58	1.26 (0.81–1.98)	0.61
Female	280	88 (31.43)	26.27–37.09		
Season					
Cold	224	132 (58.93)	52.39–65.17	505.65 (31.11–8219.78)	<0.001^***^
Hot	176	0.00	0.00–2.14		

*p < 0.05; ***p < 0.001.

### 3.2 Morphological characteristics of the egg and larva of *Haemonchus* spp. encountered in the feces of sheep

**Figure 2:**
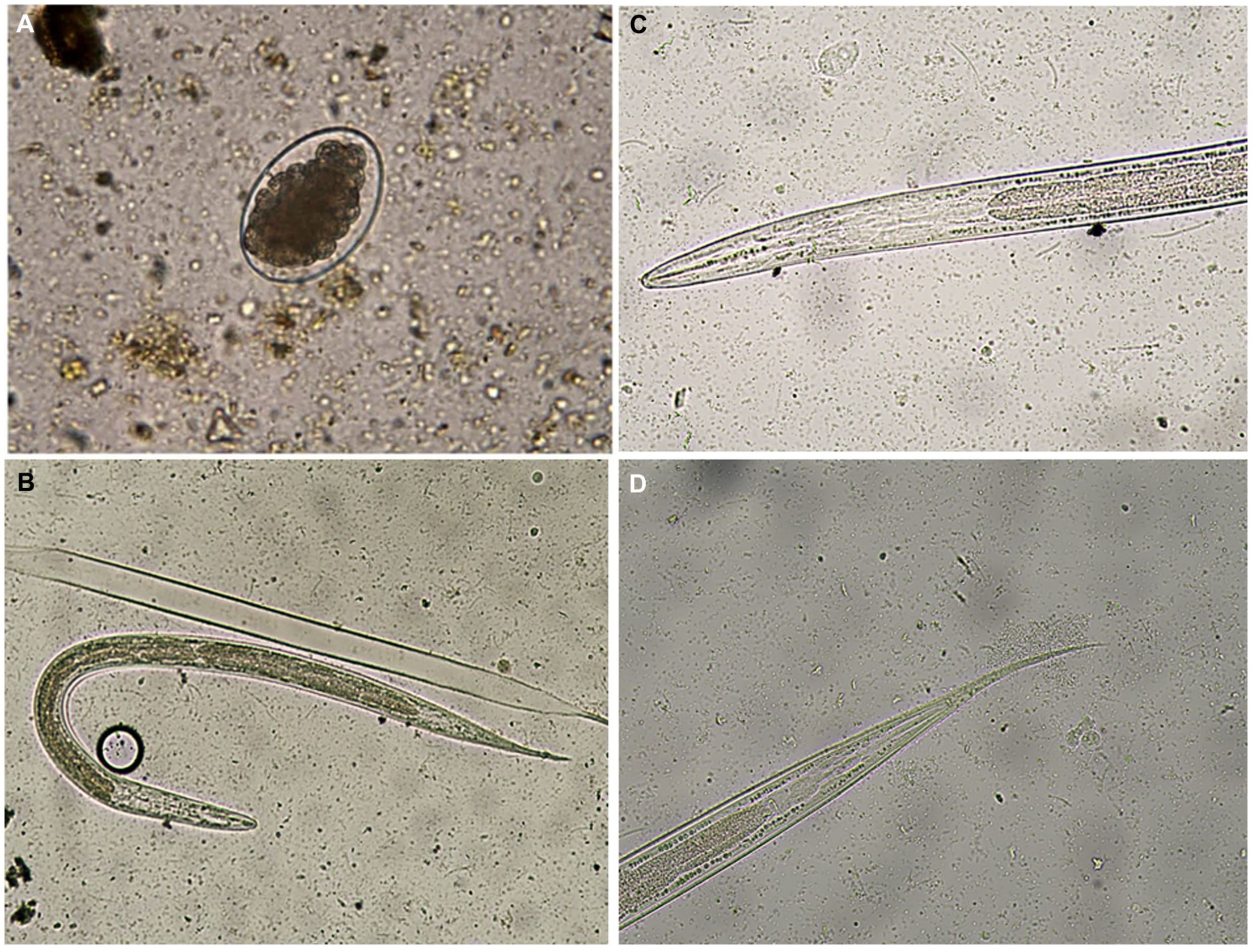
**(A)** A Haemonchus spp. egg containing the morula stage detected in the feces of sheep (400×). **(B)** *Haemonchus contortus* second-stage larva recovered from sheep fecal culture (100×). **(C)** The anterior region of a *Haemonchus contortus* second-stage larva showing a rounded head and rhabditiform esophagus (400×). **(D)** The posterior region of a *Haemonchus* contortus second-stage larva showing a pointed tail with a medium sheath (400×).

As shown in Figure 2A, *Haemonchus* spp. eggs were identified as medium-sized eggs, with oval barrel-shaped side walls. Their shells were thin with one pole rounded and the other pointed with a morula stage (16–32 cells). In relation to larvae, they had a bullet-shaped head, with a medium-length tail sheath ending in a fine point (Figures 2B–D).

### 3.3 Molecular identification of *Haemonchus* spp.

**Figure 3:**
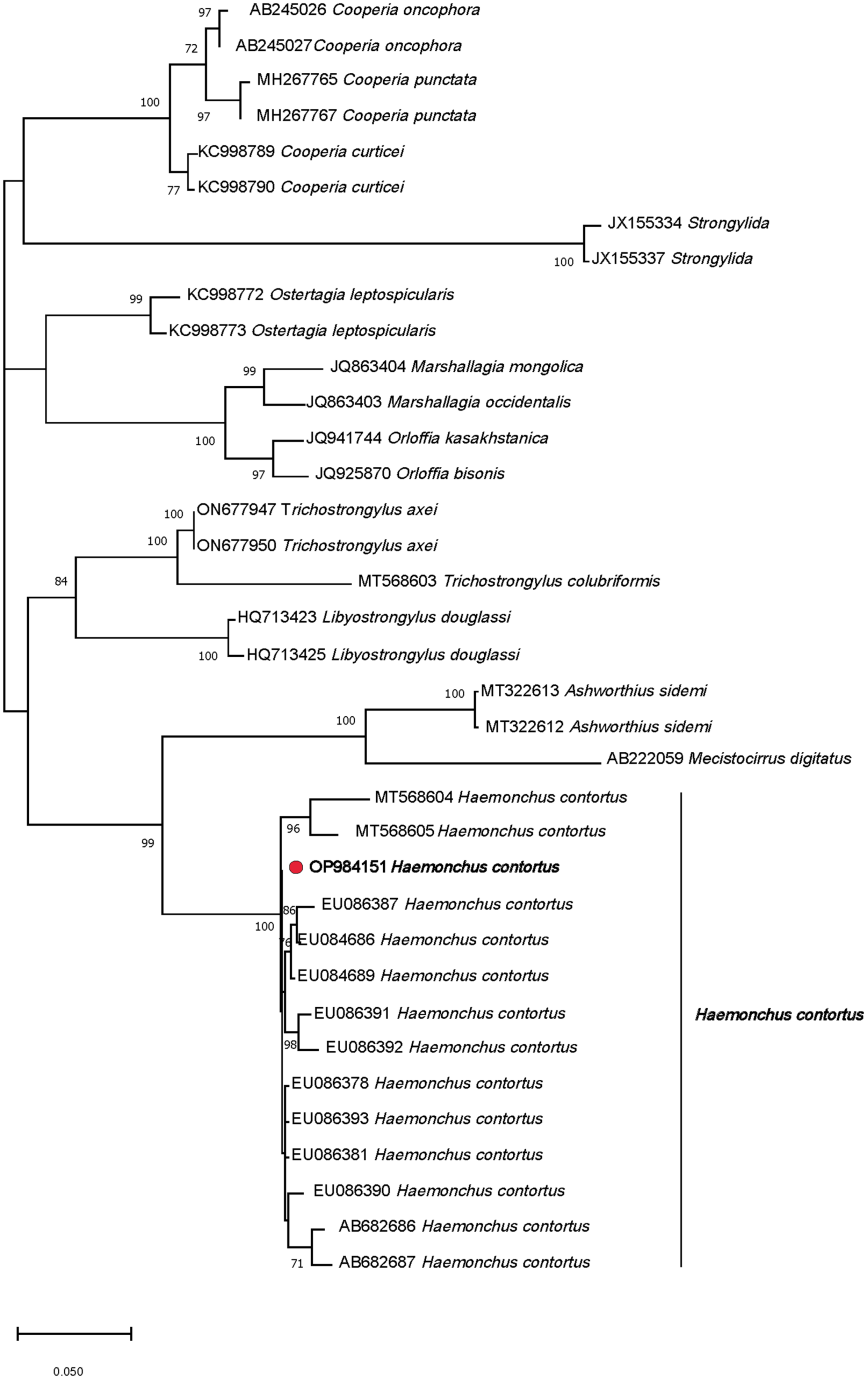
Phylogenetic tree of the *Haemonchus contortus* strain (red circle) recovered from sheep fecal culture.

A total of 240 morphologically distinguishable third-stage larvae were processed in four pools (60 larvae for each pool), which then underwent DNA extraction, amplification, and sequencing. BLASTn analysis revealed 100% homologies for all of them with *H. contortus* sequences (accession numbers, LS997564 or ON113484). Phylogenetic analysis of the PCR products confirmed the identification of *H. contortus*. In this respect, the obtained sequence revealed a strain located in the main clade that had two branches; the first subclade included the genus *Ashworthius*, with 99% bootstrap support. Although the genus *Haemonchus* occupied an independent subclade, the recovered strain (red circle) OP984151 *H. contortus* VMS5 showed 100% similarity with the established genes of *Haemonchus* (Figure 3).

## 4 Discussion

The current study has unveiled intriguing parasitological and molecular insights into *Haemonchus* infection among sheep in Upper Egypt, along with an exploration of the primary risk factors linked to this infection. Furthermore, the study conducted a national-level review of haemonchosis prevalence, with detection rates ranging from 7.00 to 49.00% (Table 1). In the present study, the overall prevalence of *Haemonchus* infection was 33.00% (132 of 400), which was one of the highest values for Upper Egypt and, clinically, the positive animals displayed symptoms such as emaciation, anemia, reduced appetite, and diarrhea. Taken into account, very few molecular studies have been conducted at a national level and the majority of the previous studies were based on macroscopical methods for the identification of the parasite. At a national level, *Haemonchus* spp. were microscopically identified among 30.00% of the examined animals in a previous study in Egypt (24), which is slightly lower than our present results. In other nearby countries, a previous study (52) in Tunisia obtained a lower prevalence rate of 17.00%. Similar results (34.51%) were reported in a previous study in Iraq (53). By contrast, a study in Sweden (54) found a higher prevalence rate of 56.00% using microscopy and 72.00% through ddPCR. Another Swedish study (55) identified *H. contortus* in 37.00% of the examined sheep. Furthermore (56), a study in Rwanda recorded a prevalence rate of 83.40% for *Haemonchus* spp. These variations might be attributed to differences in climatic conditions, geographical locations, management practices, and the methodologies employed for pathogen detection (14, 15).

In this study, age was recorded as a significant factor affecting *Haemonchus* infection in sheep. In this respect, animals less than 2 years of age had a higher infection rate than that of older animals (*p* < 0.05). These findings agree with previous studies (53, 56, 57) in which the young animals were more vulnerable to infection than adults. The higher infection in young animals may be related to undeveloped active immunity at a younger age (58). In relation to the sex of the inspected sheep, the study exhibited no significant deviation (*p* > 0.05) between the sex of the infected animals, with prevalence rates of 36.70 and 31.40% in male and female sheep, respectively. This slight percentage difference may be attributed to the fact that most females are kept indoors for breeding under careful and clean management, while most males are allowed to graze outdoors, potentially exposing them to a higher risk of infection. Similar findings were reported by Aga et al. (57) in a previous study in Western Oromiya, Ethiopia. On the other hand, our results are not consistent with other previous studies (24, 53) that found that females were more susceptible than males. Another previous study (56) found a similar infection rate in male and female sheep. The seasonal variation was reported as another significant risk factor associated with *Haemonchus* infection in the examined sheep, with a rate of 58.90% (132 of 224) in cold or winter; no infections were identified in summer (*p* < 0.05). The obtained results agree with a previous study (55) that mentioned that the fecal egg counts of ewes decreases to insignificant levels in summer, and the infections tended to rise through autumn and were highest in lambs that persisted on the farms at the end of winter. Moreover, a previous study (59) concluded that *Haemonchus* spp. have a distinctive feature, which refers to inhibited development (hypobiosis). Seasonal changes have been planned to be the main factor of hypobiosis. The feature is initiated when occasions for the spreading of the larvae are limited, which is a parasitic variation in cold weather. Additionally, hypobiosis can occur in high temperature conditions during the dry season (i.e., low humidity conditions). Furthermore, it was mentioned that, in Mediterranean climate regions, including Egypt, the outlines of *Haemonchus* spp. infection in small ruminants is frequently biphasic, with the highest rates occurring from early autumn to early winter and also from late spring to early summer (59).

The diagnosis of gastrointestinal nematodes in sheep is commonly accomplished through stool analysis, with the identification of nematode species primarily relying on the morphology of eggs and larval stages found in feces. Importantly, many species of strongyle egg, such as *Haemonchus*, *Trichostrongylus*, *Teladorsagia*, *Cooperia*, and *Bunostomum*, are similar in size and shape (60). Consequently, they cannot be easily distinguished at the genus level. Therefore, it is clear that combining fecal culture and the molecular investigation of the third-stage larva (L3) is essential for the specific identification of gastrointestinal nematodes in livestock (61–63). The molecular detection of circulating species of parasites in a specific area is invaluable for understanding the disease’s epidemiology and implementing effective control measures to combat the infection. It is worth noting that the most prevalent molecular markers include cytochrome c oxidase subunit I (cox1) of mitochondria, NADH dehydrogenase subunit, and the internal transcribed spacers (ITS1 and ITS2) of ribosomal DNA, which have consistently served as genetic markers for identifying the strongyle species in domestic animals (62–66). In the present study, the phylogenetic analysis of PCR products from larval DNA revealed that the most prevalent nematode causing GIP problems in sheep flocks in Assiut was *H. contortus* (61, 67, 68).

## 5 Conclusion

This study addressed the prevalence of haemonchosis in sheep from Upper Egypt, identifying one of the highest levels recorded in the region. It was observed that some individual variable factors, such as age and the time of the year, appear to influence the risk of *H. contortus* infestation. Updated knowledge of these aspects can increase the efficiency of diagnostic and control methods, thereby reducing the associated risks of this disease. Our current study highlights the substantial advantages of combining morphological and molecular techniques for haemonchosis detection in sheep. This approach has facilitated the identification of the *H. contortus* species in the studied samples. This species demonstrates a high biotic potential, leading to rapidly increasing parasitic loads that impact health and limit productivity. In terms of the costs associated with control through chemical treatments, significant direct and indirect losses occur, contributing to a decline in sheep productivity, profitability, and sustainability. Considering these findings, we recommend that future studies delve into the extensive prevalence of nematode species on a broader scale in Egypt. This approach is crucial for the epidemiological study and management of *Haemonchus* spp.

## Data availability statement

The original contributions presented in the study are publicly available. This data can be found at: https://www.ncbi.nlm.nih.gov/nuccore/; OP984151.

## Ethics statement

The animal studies were approved by research ethical committee of the Faculty of Veterinary Medicine, Assiut University (Approval number 06/2023/0096). The studies were conducted in accordance with the local legislation and institutional requirements. Written informed consent was obtained from the owners for the participation of their animals in this study.

## Author contributions

SM: Conceptualization, Formal analysis, Funding acquisition, Investigation, Methodology, Resources, Supervision, Validation, Writing – original draft, Writing – review & editing. AD: Conceptualization, Formal analysis, Investigation, Project administration, Software, Supervision, Validation, Visualization, Writing – original draft, Writing – review & editing. ER-Á: Data curation, Formal analysis, Funding acquisition, Methodology, Validation, Visualization, Writing – review & editing. FA-A: Conceptualization, Formal analysis, Funding acquisition, Investigation, Methodology, Software, Validation, Visualization, Writing – original draft, Writing – review & editing. FO: Conceptualization, Methodology, Software, Supervision, Validation, Visualization, Writing – original draft, Writing – review & editing. AG: Data curation, Formal analysis, Investigation, Resources, Supervision, Validation, Visualization, Writing – original draft, Writing – review & editing. AF: Data curation, Funding acquisition, Validation, Writing – original draft. DS: Data curation, Software, Validation, Writing – original draft. ME-K: Funding acquisition, Resources, Software, Validation, Writing – original draft. DB-B: Software, Validation, Writing – original draft. AA: Investigation, Writing – original draft. EE: Conceptualization, Data curation, Formal analysis, Funding acquisition, Investigation, Resources, Software, Validation, Visualization, Writing – original draft, Writing – review & editing.

## Footnotes

^1^ https://blast.ncbi.nlm.nih.gov/Blast.cgi
