# Supplementary material for: Molecular identification of Haemonchus contortus in sheep from Upper Egypt
Source: Front Vet Sci. 2024 Feb 12;10:1327424. doi: 10.3389/fvets.2023.1327424 (PMC10894989; doi:10.3389/fvets.2023.1327424)
Supplement: Supplementary file 1 [file Data_Sheet_1.ZIP › Supplementary Tables/Supplementary Table 1 Haemonchus MS.docx]

**Supplementary Table 1** Oligonucleotides used for the molecular identification of *Haemonchus* *contortus* in this study.

| **Reference** | **Length of amplified product** | **Primer sequence**  **(5'-3')** | **Target** |
| --- | --- | --- | --- |
| (42) | About 900 bp | CGCGAATRGCTCATTACAACAGC | *Nematode 18S rDNA* |
|  |  | GGGCGGTATCTGATCGCC |  |
