# Supplementary material for: Molecular identification of Haemonchus contortus in sheep from Upper Egypt
Source: Front Vet Sci. 2024 Feb 12;10:1327424. doi: 10.3389/fvets.2023.1327424 (PMC10894989; doi:10.3389/fvets.2023.1327424)
Supplement: Supplementary file 1 [file Data_Sheet_1.ZIP › Supplementary Tables/Supplementary Table 2 Haemonchus MS.docx]

**Supplementary Table 2.** PCR cycling conditions used for the molecular identification of the *Haemonchus* *contortus* investigated in this study.

|  |  | **Temperature and time** | | | | |  |  |  |
| --- | --- | --- | --- | --- | --- | --- | --- | --- | --- |
| **Target organism** | **Locus** | | **Initial denaturation** | **Denaturation** | **Annealing** | **Extension** | **No. cycles** | **Final extension** | **Reference** |
| *Haemonchus* spp. | *Nematode*  *18S rDNA* | | 94°C 5 min | 94°C 30 s | 54°C 40 s | 72°C 1 min | 35 | 72°C 10 min | (42) |
|  |  | |  |  |  |  |  |  |  |
